# Behavioral Data Analysis of Robot-Assisted Autism Spectrum Disorder (ASD) Interventions Based on Lattice Computing Techniques

**DOI:** 10.3390/s22020621

**Published:** 2022-01-14

**Authors:** Chris Lytridis, Vassilis G. Kaburlasos, Christos Bazinas, George A. Papakostas, George Sidiropoulos, Vasiliki-Aliki Nikopoulou, Vasiliki Holeva, Maria Papadopoulou, Athanasios Evangeliou

**Affiliations:** 1 HUMAIN-Lab, International Hellenic University (IHU), 65404 Kavala, Greece; lytridic@teiemt.gr (C.L.); chrbazi@teiemt.gr (C.B.); georsidi@teiemt.gr (G.S.); 2 MLV Research Group, Department of Computer Science, International Hellenic University (IHU), 65404 Kavala, Greece; gpapak@teiemt.gr; 3 1st Psychiatric Clinic, Papageorgiou General Hospital, Aristotle University of Thessaloniki, 56403 Thessaloniki, Greece; v.a.nikopoulou@gmail.com (V.-A.N.); vholeva@yahoo.gr (V.H.); 4 4th Department of Pediatrics, Papageorgiou General Hospital, Aristotle University of Thessaloniki, 56403 Thessaloniki, Greece; mtpapado@gmail.com (M.P.); aeevange@auth.gr (A.E.)

**Keywords:** autism spectrum disorder (ASD), human–social robot interaction, machine learning, special education

## Abstract

Recent years have witnessed the proliferation of social robots in various domains including special education. However, specialized tools to assess their effect on human behavior, as well as to holistically design social robot applications, are often missing. In response, this work presents novel tools for analysis of human behavior data regarding robot-assisted special education. The objectives include, first, an understanding of human behavior in response to an array of robot actions and, second, an improved intervention design based on suitable mathematical instruments. To achieve these objectives, Lattice Computing (LC) models in conjunction with machine learning techniques have been employed to construct a representation of a child’s behavioral state. Using data collected during real-world robot-assisted interventions with children diagnosed with Autism Spectrum Disorder (ASD) and the aforementioned behavioral state representation, time series of behavioral states were constructed. The paper then investigates the causal relationship between specific robot actions and the observed child behavioral states in order to determine how the different interaction modalities of the social robot affected the child’s behavior.

## 1. Introduction

One of the areas where social robots have been applied in recent years is education, as well as special education for children. The main reasons behind the popularity of such applications is the motivation and the guidance that social robots provide [1,2].

In special education, more specifically regarding Autism Spectrum Disorder (ASD) interventions, the ability of robots to improve the social and communication skills of children has been confirmed [3]. Numerous studies have demonstrated the use of social robots toward improving social skills such as joint attention [4,5,6]. Safety issues have also been considered [7]. The typical approach for an effective interaction with a human is to ensure that, first, the robot attracts a child’s attention, followed by, second, a high level of engagement throughout an educational session. Consequently, the robot pursues an effective interaction by timely issuing visual (e.g., movements, blinking lights, facial expressions) or auditory (e.g., speech, sounds, music) stimuli triggered by feedback sensory data techniques. The types of interaction engaged depend on factors such as a child’s language skills [8].

Measuring a child’s engagement is critical for maintaining the interaction of a social robot with a child. Various techniques have been proposed in the literature for measuring engagement [9] including machine learning techniques [10]. In an early investigation of child behavior triggered by robot actions, the average behavior times for contact and gaze were used to evaluate the interaction duration [11]. In this work, it has been shown that child actions generally last longer when the child interacts with a robot as opposed to a toy. In Ref. [12] a simulation study was presented, where a learning framework was used to train a robot according to a human supervisor’s corrective actions to a given child state. In Ref. [13], the objective was to infer the engagement of one of the participants using cues from other participants present, in a collaborative task with an NAO robot. Such cues include head nods, visual focus of attention, utterances, gaze, etc. In Ref. [14], the engagement was measured in terms of the time to task completion and/or the total duration of interaction; furthermore, cameras are used to analyze the human–robot interaction sessions as well as to calculate engagement measures. In Ref. [15] the comparative effect of pairs of visual, motion and audio stimuli on a child’s attention was measured based on eye contact measurements by the NAO robot in real-time during child–robot interaction. Software-based annotated behaviors that indicate social attention, as well as social responsiveness, were used in [16] to construct an engagement index in order to quantify the effectiveness of robot actions based on coded video data. Video recordings were used in [8] to study spontaneous child responses elicited from robot actions; in particular, child behaviors were annotated by human coders; moreover, the frequency of the various observed interaction types was both recorded and analyzed. In Ref. [17] fixation time as well as gaze transitions from eye-tracking data were used to compare differences in elicited attention between typically developing children and children with ASD. In another study [18], the data collected from the sensory system were mapped onto the child’s identified behaviors based on training and validation sets of child–robot interactions annotated by experts. In [19], a system was presented which combines data from body pose, gaze and gestures to determine whether a human intends to interact. The effect of robot feedback on children with ASD in an imitation task was investigated in [20], where the authors measured the number of prompts that need to be issued by the robot when an imitation is not executed correctly; moreover, the accuracy of the imitation was automatically estimated by a Kinect motion sensor. Furthermore, the work in [21] used a deep learning method to estimate engagement based on the child’s pose as determined by recordings from four cameras. The effect of visual stimuli on child attention was explored in [22]. The authors examined eye contact occurrences in response to different types of LED activation patterns of a NAO robot.

In a recent study [23], a post-intervention analysis on multi-modal data collected during learning activities involving a humanoid robot and two children was presented. The authors extracted engagement-related features from video, audio and log data in order to establish correspondences between clustered pairs of students and their corresponding behavior patterns and learning labels. The objective of the work was to help the robot to determine the time of effective intervention as well as the type of the most beneficial behavior to be induced in the users. Ad hoc feature extraction has also been considered for the estimation in children engagement [24]. With this method, machine learning techniques are employed to determine from multi-modal data whether the child is engaged or not, based on manual annotations by experts. Deep convolutional networks were used for engagement estimations in [25]. In this study, strictly posed features were used for typically developing children and children with ASD. The data in this work were also manually annotated by humans according to a set of instructions provided by an expert psychologist. Additionally, in [26] the authors examined how participants of a dance-based activity responded to either positive or negative feedback by the robot, delivered in the form of speech. The study presented in [27] investigated latencies in shifting attention in response to robot-initiated stimuli in children with ASD. The study used two NAO robots to produce visual, speech, motion and social stimuli and used the child’s gaze to determine attention shift and the difference between the time at which the stimulus was produced and the time at which attention was established. In Ref. [28], attention assessment was achieved by calculating an attention score using face attention and joint attention scores and sound responses. Head pose and sound data were collected using an external mobile phone camera attached to the body of a NAO robot. On the other hand, emotion detection was performed using NAO’s head camera. Subsequent interactions were tuned based on previous interaction results. The effectiveness of light color variation, auditory and motion stimuli produced by a robot during robot-assisted ASD therapy was studied in [29]. Joint attention and eye contact duration were used to assess how robot stimuli affect child behavior. The results indicate that the effectiveness of the stimulus depends on the autism category.

As it can be seen from the above, typical studies dealing with engagement rely heavily on detecting and interpreting human movements. However, not all human movements are meaningful in human–robot interaction; therefore, a vision-based system must be able to distinguish between movements that describe behavior from those that describe emotion or intention, etc. In addition, it is not always clear whether a certain movement is in response to a specific stimulus. Hence, for dependable analysis and design, a systematic study is necessary regarding how a robot’s actions may trigger a child’s behavior. Moreover, effective information representation techniques, including semantics, are promising. Research in this field often considers cumulative measurements regarding engagement over the course of a human–robot interaction session such as measuring the total duration of gaze with the robot. However, few studies examine either the specific effect of combined interaction modalities or the association of a robot action(s) to a child’s response. On the other hand, they mostly assume human annotations of child behaviors rather than automatic (machine) annotations. There is a shortage of mathematically sound tools for the analysis of human behavior during interaction with a social robot toward a rigorous design of an effective interaction with a human. Finally, it is common for studies that investigate the effect of various stimuli to implement purpose-made scenarios to collect measurements and conduct the analysis, instead of collecting data from existing therapeutic protocols.

This work addresses the aforementioned technical shortcomings. The two main contributions of the present paper are summarized as follows: (1) the development of novel computational instruments for the representation of behavioral states without the need for manual behavior annotation, and (2) a novel data analysis methodology for providing a better insight into induced child behavior in response to robot actions. As far as the computational instruments are concerned, novel data representation structures and processing tools have been developed so that non-numerical data that represent semantics in human–robot interaction can be practically processed. In addition, these lattice computing-based computational tools have been utilized in conjunction with an existing clustering algorithm in order to induce data-driven behavioral states, instead of pursuing the classical approach of classifying the observed data to pre-determined behaviors. On the other hand, the data analysis conducted using the aforementioned computational instruments reveals the relationship between robot events and child responses. The proposed methodology allows psychologists to determine the significance and effectiveness of the various robotic stimuli in a child’s engagement. Analyses can be conducted at various levels either per activity or per child.

The proposed techniques have been developed collaboratively by computer scientists and system engineers with the advice of medical doctors as well as of clinical psychologists. Based on real-world data acquired in a clinical environment by sensors mounted on a humanoid social robot, namely NAO, the idea here is to present a proof of concept regarding novel tools toward (a) the quantitative evaluation of child–robot interactions during therapeutic interventions and (b) the more effective design of future intervention protocols. Only human face data are considered in this work for behavioral state representation. However, due to the inherently modular mathematical techniques considered here, enhancements toward including additional types of data are straightforward as well as sound. It is worth noting that in this work, assessments of the causal relationship between stimuli and child behavior were conducted by examining an existing robot-assisted therapeutic protocol in a real-world therapeutic setting, as opposed to child–robot interaction activities specifically designed for data collection purposes.

The approach proposed here is inspired from “structuralism” in general science [30] including psychology [31], neurology [32] and biology [33]. In particular, human behavior here is represented as a time series of “segments”. The latter are information *granules*, interpreted as *states* (of a human), induced from recorded data by computer clustering techniques. It is understood that the total number of clusters to be calculated by a computer as well as the meaning of each cluster is subjective. However, such assumptions may result in a computer-supported analysis of human behavior toward an effective human–robot interaction design. In conclusion, a child’s behavior is represented here by a time series of states. Note that human–robot interaction may involve more complex data than merely real number measurements; for instance, it may involve structural data representing human body posture and sets of features, among others. In the latter context, a novel mathematical background for rigorous analysis is required that may accommodate disparate types of data as explained next.

Lately, the Lattice Computing (LC) information processing paradigm has been proposed for modeling in Cyber–Physical System (CPS) applications, including human–robot interaction applications [34]. Recall that an LC has been defined as “an evolving collection of tools and methodologies that process lattice ordered data including logic values, numbers, sets, symbols, graphs, etc.”. During its interaction with humans, a social robot is driven by software; the latter implements a mathematical model. Note that, typically, a model is developed in the Euclidean space, and it processes real numbers stemming from electronic sensors measurements. However, when humans are interacting with social robots, non-numerical data also emerge that represent semantics. An LC model has the capacity to compute with semantics represented by a lattice partial order relation; moreover, an LC model can compute with big numerical data as well as with non-numerical data such as trees data structures, probability spaces, etc. A number of applications have demonstrated the efficiency of LC models to represent various types of data in computational intelligence applications efficiently as well as effectively [35,36,37].

The present paper is structured as follows. Section 2 describes useful data representations as well as mathematical instruments. Section 3 details both data acquisition and pre-processing. Section 4 presents the proposed tools as well as their application results. Finally, Section 5 concludes this paper by discussing the contribution of this work and delineating potential future work extensions.

## 2. Data Representation and the Mathematical Instruments

This section outlines the data representation as well as novel mathematical instruments used below.

### 2.1. Face Representation

**Figure 1:**
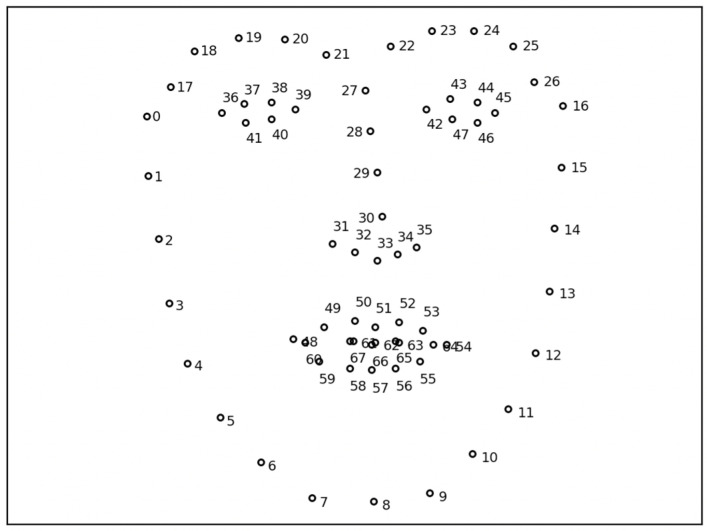
Detection of the 68 facial landmarks by the OpenFace library in a face image collected by the robot’s camera.

The OpenFace library [38] was used to detect 68 key points called (*facial*) *landmarks* on the human face, as shown in Figure 1, and return their coordinates. Given an image of a human face, this widely used machine vision library is pre-trained to detect and track specific keypoints in real time. The limitation is that the entire face has to be clearly visible to the camera for the 68 landmarks to be detected. This means that detection can fail if the face is turned above a certain threshold or if it is far from the camera.

**Figure 2:**
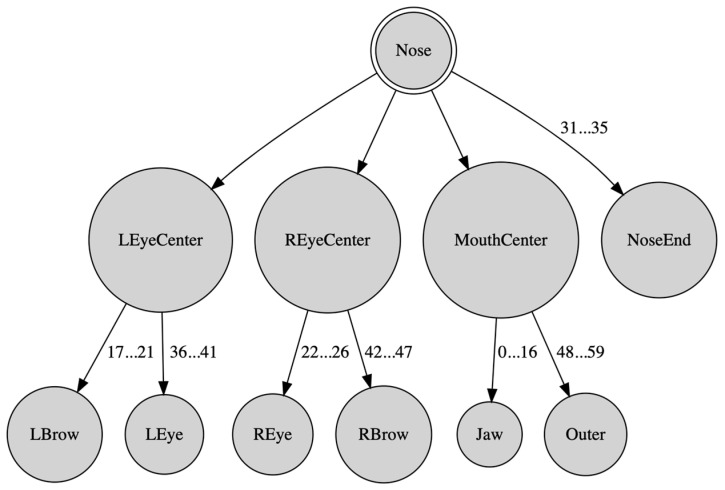
Facial features represented by a tree data structure.

In this work, a human face was represented as a tree data structure induced from the facial landmarks, as presented in [39,40]. In this representation, selected landmarks were utilized to consequently construct a tree hierarchy of features, which was then used as a computational primitive in the context of lattice computing. More specifically, except for the root node, every tree node corresponded to a vector, represented by polar coordinates, connecting its parent node to a point; hence, each node was characterized by two values, namely the corresponding vector’s length *r* and orientation *φ*. The tree data structure is illustrated in Figure 2, where, for concise visualization reasons, related nodes are grouped together. Each tree node is a facial feature, computed using the original landmark data. The facial features used to construct this tree representation were chosen because of their significance in constituting the human face. The polar coordinates for each node constitute the spatial arrangement of the nodes and their relation to each other.

Note that the tree in Figure 2 consists of three levels: The root of the tree, the Nose, is defined by two points, namely the top and tip (of the Nose). The next tree level includes nodes noted as *primary features*, which are computed as follows. The first two primary node features are the vectors connecting the top of the Nose to the center of the left/right eye, whose position is defined as the center of mass of the respective eye contours; the next primary node feature is the vector connecting the tip of the Nose to the center of the mouth calculated as the center of mass of the mouth’s outer contour; the last five primary nodes features are vectors connecting the tip of the Nose to the five points defining the bottom (i.e., the nostrils) of the Nose. Likewise, vectors were computed for *secondary features* that include points of the left/right eyes and brows, inner/outer mouth contours and the jaw. In total, there were 8 primary and 51 secondary features. Scale/rotation/translation invariance was achieved by defining the vector from the top to the tip of the Nose as the unit vector. Furthermore, a vector orientation was measured in radians in the range [−π, π]. An additional advantage of the proposed tree data structure is that it guarantees the anonymity of the human face it represents.

### 2.2. Distance Metrics between Trees Data Structures

In all, 59 = 8+51 primary and secondary node features of the tree data structure in Figure 2 were used, i.e., (*r_i_*,*φ_i_*) pairs of polar vector coordinates, where *i* ∈ {1, …, 59}. In particular, a pair ([*a_i_*,*b_i_*], [*c_i_*,*d_i_*]) of intervals was used with the potential to represent information granularity regarding the corresponding polar coordinates. In the special case that *a_i_* = *b_i_* = *r_i_* as well as *c_i_* = *d_i_* = *φ_i_*, *i* ∈ {1, …, 59}, the corresponding tree data structure is called *trivial tree*, and it represents a single face, whereas for *a_i_* < *b_i_* and/or *c_i_* < *d_i_*, *i* ∈ {1, …, 59}, the corresponding tree data structure is called *interval tree*, and it represents an information granule, i.e., a neighborhood of faces. To facilitate implementation of various algorithms below, the 59 tree nodes in Figure 2 were renumbered top-down and from left to right by the numbers from 1 to 59, respectively; the tree root was given number 0.

Previous work has used the abovementioned tree data structure as an instrument for vector feature extraction [39]. In contrast, the present paper considers a tree’s hierarchical structure as follows. Since secondary features stem from primary ones, it was assumed that any calculation that involves a primary feature must take into account the corresponding children secondary features as well, albeit to a different degree. In the latter manner, the hierarchical tree data structure was considered. Hence, the distance *D*(*T_a_*,*T_b_*) between two corresponding branches *T_a_* and *T_b_*, in two different trees, was calculated, based on LC, as follows:

(1) $$\left(T_{a},T_{b}\right)=d_{p}\left(\left[a_{p},b_{0}\right],\left[c_{p},d_{p}\right]\right)+k_{c}\left[D_{1}\left(T_{a_{1}},T_{b_{1}}\right)+\cdots +D_{C}\left(T_{a_{C}},T_{b_{C}}\right)\right],$$

Equation (1) was applied on a tree data structure incrementally from the leaves to the root. Specifically, for two whole tree data structures *T_a_* and *T_b_* of Figure 2 it follows

(2) $$D\left(T_{a},T_{b}\right)=D_{1}\left(T_{a_{1}},T_{b_{1}}\right)+D_{2}\left(T_{a_{2}},T_{b_{2}}\right)+D_{3}\left(T_{a_{3}},T_{b_{3}}\right)+D_{4}\left(T_{a_{4}},T_{b_{4}}\right)+\;D_{5}\left(T_{a_{5}},T_{b_{5}}\right)+D_{6}\left(T_{a_{6}},T_{b_{6}}\right)+D_{7}\left(T_{a_{7}},T_{b_{7}}\right)+D_{8}\left(T_{a_{8}},T_{b_{8}}\right),$$

because the distance $d_{p}\left(\left[a_{p},b_{0}\right],\left[c_{p},d_{p}\right]\right)$ between the normalized roots (i.e., the Nose) of two different trees *T_a_* and *T_b_* was zero; moreover, without loss of generality, it was assumed that *k_c_* = 1. In particular, the terms *D*_1_(.,.) … *D*_8_(.,.) in Equation (2) were computed as follows.

$$D_{1}\left(T_{a_{1}},T_{b_{1}}\right)=d_{1}\left(\left[a_{1},b_{1}\right],\left[c_{1},d_{1}\right]\right)+k_{1}\left[d_{9}\left(\left[a_{9},b_{9}\right],\left[c_{9},d_{9}\right]\right)+\cdots +d_{19}\left(\left[a_{19},b_{19}\right],\left[c_{19},d_{19}\right]\right)\right]\;D_{2}\left(T_{a_{2}},T_{b_{2}}\right)=d_{2}\left(\left[a_{2},b_{2}\right],\left[c_{2},d_{2}\right]\right)+k_{2}\left[d_{20}\left(\left[a_{20},b_{20}\right],\left[c_{20},d_{20}\right]\right)+\cdots +d_{30}\left(\left[a_{30},b_{30}\right],\left[c_{30},d_{30}\right]\right)\right]\;D_{3}\left(T_{a_{3}},T_{b_{3}}\right)=d_{3}\left(\left[a_{3},b_{3}\right],\left[c_{3},d_{3}\right]\right)+k_{3}\left[d_{31}\left(\left[a_{31},b_{31}\right],\left[c_{31},d_{31}\right]\right)+\cdots +d_{59}\left(\left[a_{59},b_{59}\right],\left[c_{59},d_{59}\right]\right)\right]\;D_{4}\left(T_{a_{4}},T_{b_{4}}\right)=d_{4}\left(\left[a_{4},b_{4}\right],\left[c_{4},d_{4}\right]\right)\;D_{5}\left(T_{a_{5}},T_{b_{5}}\right)=d_{5}\left(\left[a_{5},b_{5}\right],\left[c_{5},d_{5}\right]\right)\;D_{6}\left(T_{a_{6}},T_{b_{6}}\right)=d_{4}\left(\left[a_{6},b_{6}\right],\left[c_{6},d_{6}\right]\right)\;D_{7}\left(T_{a_{7}},T_{b_{7}}\right)=d_{4}\left(\left[a_{7},b_{7}\right],\left[c_{7},d_{7}\right]\right)\;D_{8}\left(T_{a_{8}},T_{b_{8}}\right)=d_{8}\left(\left[a_{8},b_{8}\right],\left[c_{8},d_{8}\right]\right)$$

Note that that Equations *D*_4_(.,.), …, *D*_8_(.,.) stem from Equation (1) based on the fact that secondary feature nodes do not have children. Moreover, a distance $d_{i}$(.,.), *i* ∈ {1, …, 59} is given by

(3) $$d_{i}\left(\left[a,b\right],\left[c,e\right]\right)=\left[v_{i}\left(\theta_{i}\left(a\wedge c\right)\right)-v_{i}\left(\theta_{i}\left(\alpha \vee c\right)\right)\right]+\left[v_{i}\left(b\vee e\right)-v_{i}\left(b\wedge e\right)\right]$$

The type of functions *ν_i_*(.) and *θ_i_*(.) for *i* ∈ {1, …, 59} is defined by the user. In previous work, *ν_i_*(.) and *θ_i_*(.) were all assumed to be *v_i_*(*x*) *= x* and *θ_i_*(*x*) *= −x*, *i* ∈ {1, …, 59} [39]. However, in this paper, non-linearities were introduced by inserting an additional parameter *λ_i_* in a *ν_i_*(.) function as follows:

(4) $$v_{i}\left(x\right)=\lambda_{i}x,$$

(5) $$\theta_{i}\left(x\right)=\theta \left(x\right)=1-x,$$

for *i* ∈ {1, …, 59}.

A distance *D(T_a_*, *T_b_)* was calculated separately for *r* and *φ*; then, the two partial results were summed up scaled by parameters *k_r_* and *k_φ_*, respectively.

## 3. Data Acquisition and Pre-Processing

This work was carried out in the context of investigating the effectiveness of robot-assisted special education delivery compared to traditional methods. The overall study regards controlled studies involving children with either Level I ASD or specific types of learning difficulties. This paper focuses exclusively on the ASD intervention part of the project. Children participants were recruited based on a diagnosis of Level I ASD [41] at the outpatient Pediatric Neurology clinic of the 4th Department of Pediatrics of the Papageorgiou General Hospital (PGH) in Thessaloniki, Greece. The intervention protocol has been reviewed and approved by the hospital’s ethics committee. All the required informed consents and clearances have been obtained by the participants’ parents or legal guardians, prior to the interventions. All legal requirements for data collection and storage were fulfilled. Sensory data acquisition was carried out exclusively by the sensors on-board the NAO robot. The recorded data were batch-processed later, using software developed specifically for this work.

### 3.1. Data Acquisition

The *protocol* that was designed by clinical psychologists at PGH [42,43] includes 9 distinct *steps*, described in Appendix A. Each step concerns a thematic unit regarding the development of specific social and/or communication skills and includes 1 or 2 *activities*, or equivalently *scenarios*, such as symbolic games, imitation games, etc. In total, 12 activities were included in the protocol. Note that the sets of activities of any two different steps are mutually exclusive. Any therapeutic *session* was designed by clinical psychologist(s) as a series of protocol steps, according to the needs of a specific child at any given day; during a session, activities were selected “on the spot” by practitioner psychologist(s) and could be executed one or more times (*attempts*).

**Figure 3:**
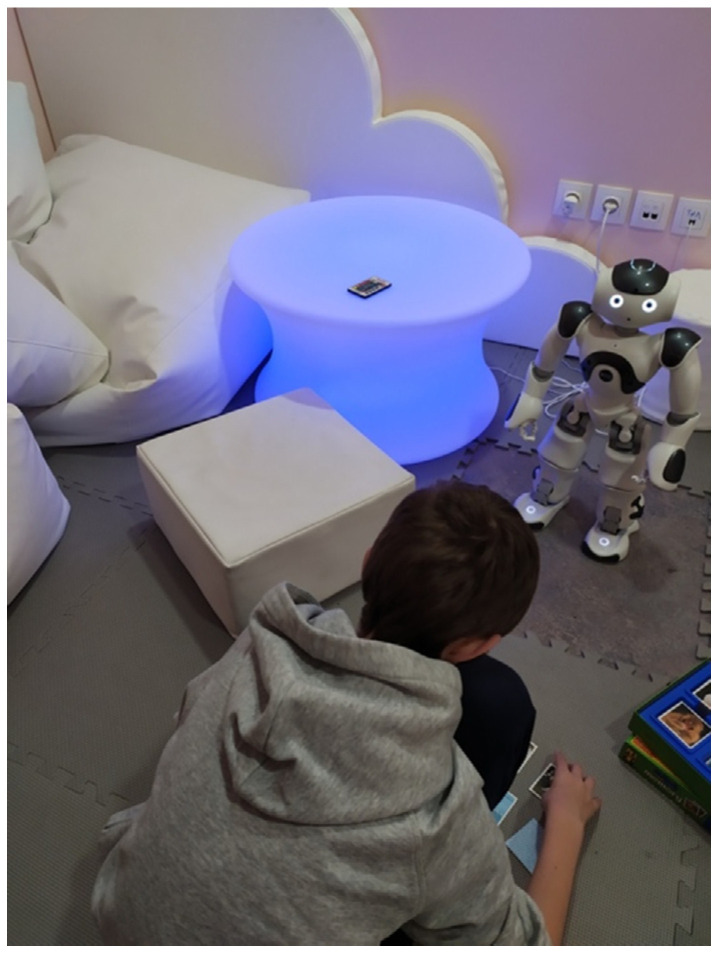
Positioning of child and robot in the therapy room during a session.

Data were acquired during all sessions at the PGH as follows. During the robot-assisted session, the robot was positioned in front of the child at a distance of approximately 1 m in order to perform the interactive activities, as shown in Figure 3.

The robot used its own sensors to log both its environment and itself. To maximize the detection capabilities of the OpenFace library, appropriate lighting conditions were ensured in the therapy room, and the robot has been programmed to move its head to track the child and maintain visual contact for as long as possible. In particular, the robot was recording a variety of anonymous data regarding the child in real-time including facial landmarks, body pose, hand gestures and eye contact as well as the volume and duration of the child’s speech. For the duration of the robot-assisted activities, the robot’s camera was continuously capturing images of the child, which were then fed to the OpenFace library in order to perform the detection of the 68 landmarks and return their (*x*,*y*) coordinates. At the same time, the robot kept track of its own actions including its body motions, posture, animations, audio playback, speech, eye LED activity, whether eye tracking was active and whether its sonar sensors detect objects in close proximity. The outset of a robot action is called an *event*. All the recorded data values (robot actions and child data) at a sampling time constitute a *frame*. Frames were acquired in this work at a sampling rate of 3 Hz, i.e., 3 frames per second. A frame is called *complete* if and only if it includes values for both the child’s face and the robot’s actions. The present work considers exclusively the frame values in Table 1, where the *state* of a child was defined by the 68 human face landmarks points. Frames where the child’s face was not recognized were discarded.

The values of each frame, which include the landmarks coordinates as well as the recorded robot actions, were stored in text files. This work considers data from seven children (aged 7 to 11 years old, six male and one female) in 82 sessions. There were 194 attempts of various activities. Only attempts including complete frames were considered; hence, 90 attempts were considered, namely *eligible attempts*. In conclusion, from the 41,911 frames recorded in 90 eligible attempts, a face was detected in 6846 complete frames due to the fact that, in many cases, a child turned away to look at either surrounding objects or the therapist or the child moved away from the robot.

### 3.2. Structured Head Pose Recognition

Head pose recognition is also important when estimating a child’s engagement. Our assumption here was that a child is engaged if and only if it orients its head directly at the robot. Therefore, head pose recognition was treated as a pattern classification problem as detailed in the following.

**Figure 4:**
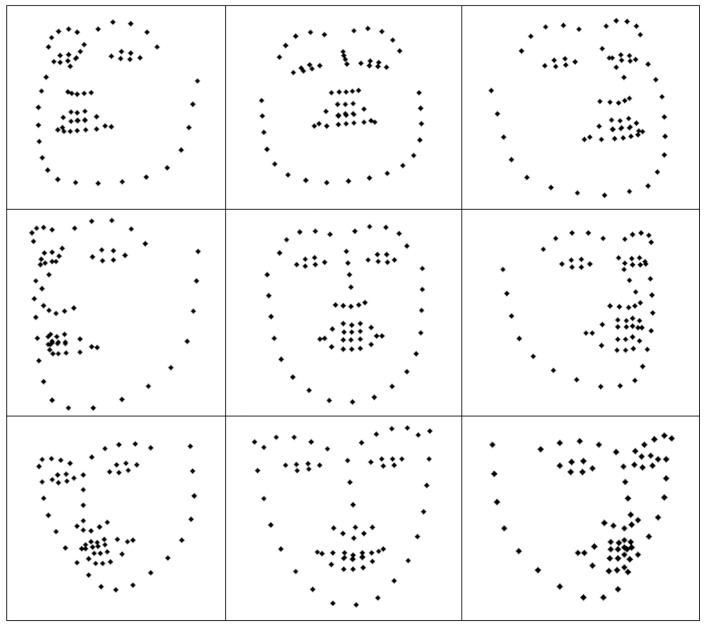
Typical examples of each of the nine head poses from the training data.

Training data were generated by recording stills of a head in nine head poses (classes), namely Front, Upper Left, Up, Upper Right, Right, Lower Right, Down, Lower Left, Left denoted by the numerals 0, 1, 2, 3, 4, 5, 6, 7, 8, respectively (Figure 4). In particular, for the class Front, three sub-classes were assumed, namely Front Left, Front Proper and Front Right. Every individual image was represented by a trivial tree as was explained in Section 2.

One hundred labeled image stills were recorded for every head pose in order to compute a total of 11 interval tree prototypes as follows. The minimum and the maximum values of *r* and *φ* for every feature at a tree node were used to calculate a pair (Δ*r_i_*, Δ*φ_i_*) of intervals, *i* ∈ {1, …, 59} per head pose. In addition, one hundred images for each of the nine basic head poses were recorded, that is, a total of 900 images were gathered for testing. More specifically, to test the robustness of the proposed method under environmental conditions encountered during ASD interventions, 900 data were collected for all combinations of the following two conditions: (1) the distance between the subject and the camera was either 40 cm or 1 m and (2) the lighting was either normal or dim.

**Figure 5:**
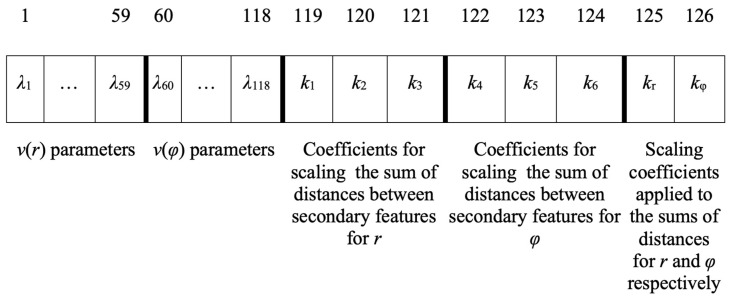
Parameters encoded in a genetic algorithm’s individual chromosome.

After the generation of the head pose prototypes, classification experiments were carried out. The objective of the classification experiments was to determine the ability of this representation in the recognition of each head pose, i.e., to correctly classify each testing image to the correct head pose class. In other words, a test image (a tree with trivial intervals) is classified to the class whose prototype (interval tree) is closest (i.e., has greater similarity). A standard 10-fold cross-validation classification was carried out on the testing data. Furthermore, parameter optimization was pursued by a genetic algorithm, in order to determine the parameters that result in greater classification rates. More specifically, a chromosome encoded the *λ_i_*, *i* ∈ {1, …, 59} parameters for *r* and the *λ_i_*, *i* ∈ {60, …, 118} parameters for *φ*; moreover, it encoded *k*_1_, *k*_2_ and *k*_3_ for *r* as well as for *φ*; finally, it encoded the *k_r_* and *k_φ_* parameters as shown in Figure 5. The Genetic Algorithm (GA) included a population of 500 individual chromosomes, each representing a specific set of parameters (Figure 5) for 50 generations with the ranges of all parameters *λ_i_*, *i* ∈ {1, …, 118}, *k_i_*, *i* ∈ {1, …, 6}, *k_r_* and *k_φ_* in the interval [0, 10].

The results for classification experiments regarding head pose estimation after parameter optimization are shown in the second column of Table 2.

These results are significant improvements compared to the results obtained in a previous work [39] shown in the third column of Table 2. In particular, previous results were obtained using the same tree structure as an instrument for feature extraction in a vector form. Table 2 demonstrates that the employment of a tree data structure improves head pose recognition. The improvement was confirmed by a paired two-sample *t*-test. There was a significant difference between the classification performance achieved by using the tree representation (M = 83; SD = 0.13) and that achieved by the vector form (M = 86; SD = 0.12); t(7) = −3.72, *p* = 0.003. This indicates that the chosen representation is a reliable instrument in determining head pose and can therefore be used for this purpose in the following analysis.

### 3.3. States Induction by Clustering

**Figure 6:**
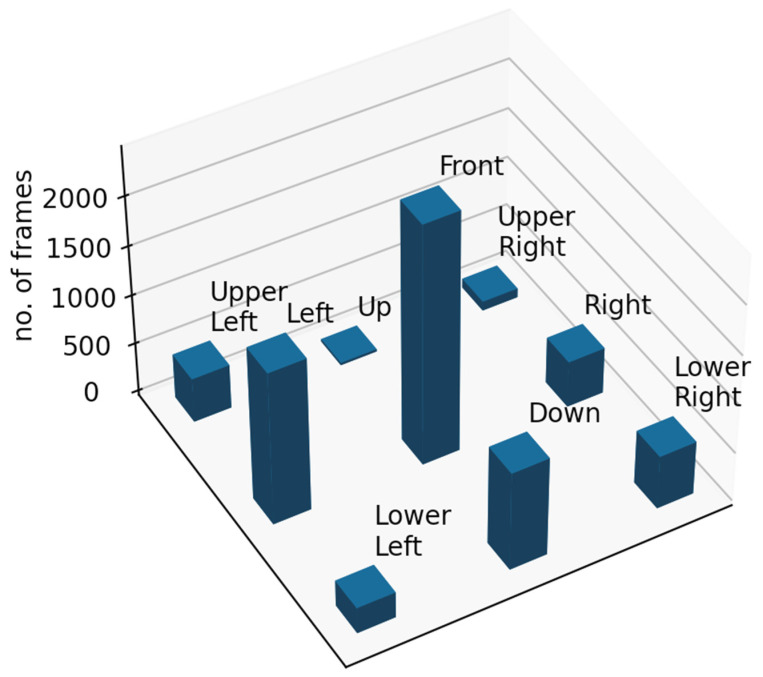
Distribution of 6846 complete frames per state.

A clustering procedure was carried out in order to induce clusters of frames, i.e., child states, from data compiled in all real-world application sessions. In particular, the *k*-means algorithm was employed using the distance of Equation (2). This distance was used instead of the Euclidean distance commonly used in the k-means algorithm because Equation (2) considers the hierarchical tree data structure. More specifically, the *k*-means algorithm was initialized using 11 trivial trees computed as the centroids (i.e., mean values) of the 11 initial interval trees prototypes described as training data above; furthermore, the parameter values used for the calculation of distances were those estimated by the genetic algorithm above. The coordinates of all complete frames of the intervention recorded text files were transformed to the tree representation described in the previous section. Therefore, a total of the 6846 trivial trees, corresponding to complete frames, were clustered. The Front Left, Front Proper and Front Right clusters were treated as one class, namely Front. Hence, nine states were computed, namely (within parentheses is shown the original number of trivial trees): Upper left (452), Up (23), Upper right (93), Left (1560), Front (2434 = 517 (Front Left) + 1750 (Front Proper) + 167 (Front Right)), Right (472), Lower left (263), Down (1004) and Lower right (545). The distribution of 6846 complete frames per class is shown in Figure 6. The shows that most frames were clustered around the cluster with the Front centroid, which is expected given that most of the time the child was facing the robot. It can also be observed that quite a few frames were centered around the Left centroid, which can be explained by the fact that during the sessions the therapist was sitting on the left side of the child, frequently attracting the child’s attention by giving instructions or asking questions.

The significance of employing clustering of the trivial trees that correspond to complete frames is that it allows data-driven creation of behavioral states, instead of merely classifying each frame as one of the training prototypes. Although clusters are centered around the mean values of the prototypes, they can contain frames that are farther away but have significant commonalities compared to other frames. This allows clusters (i.e., states) to be determined by the entirety of the available data. Clusters are also more versatile since they could allow automatic behavioral state creation in future application where more sensory stimuli are considered, and so complex behavioral units can emerge from the available data.

**Figure 7:**
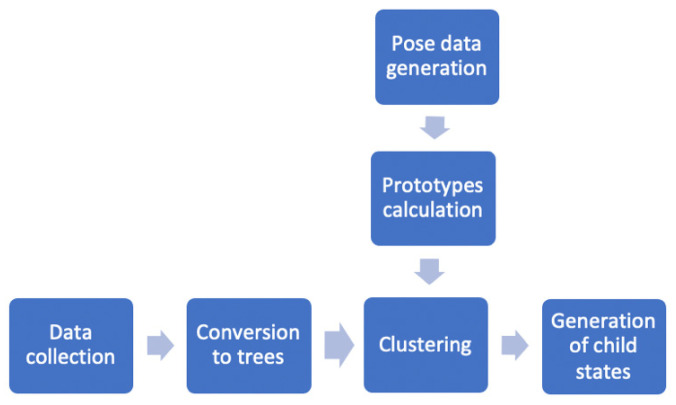
Process of determining the child states based on the collected data.

Figure 7 illustrates the overall process of creating the child states from the raw data collected by the robot during the sessions.

## 4. Tools for Behavioral Data Analysis

Using the instruments above, four tools were developed, presented one per sub-section below. In addition, note that four different robot *modalities* were considered, namely (a) Animation, (b) Sound, (c) LEDs and (d) Speaking. In particular, “Animation” typically involves dancing as well as various gestures; “Sound” typically involves music as well as various sound effects; “LEDs” typically involves photo-rhythmic eye patterns; and “Speaking” typically involves animated speech or speaking with robot movements in tune. Each modality was implemented by one or more robot *actions*. The corresponding child responses were recorded.

### 4.1. Relationship between Robot Actions and Child States

A numerical analysis was carried out on the set of complete frames. In addition to understanding child behavior, the objective, given a specific robot modality, was to calculate a probability distribution function over the set of child states toward designing a session that attracts a child’s attention and implements props to maintain engagement and develop social attention profiles.

**Figure 8:**
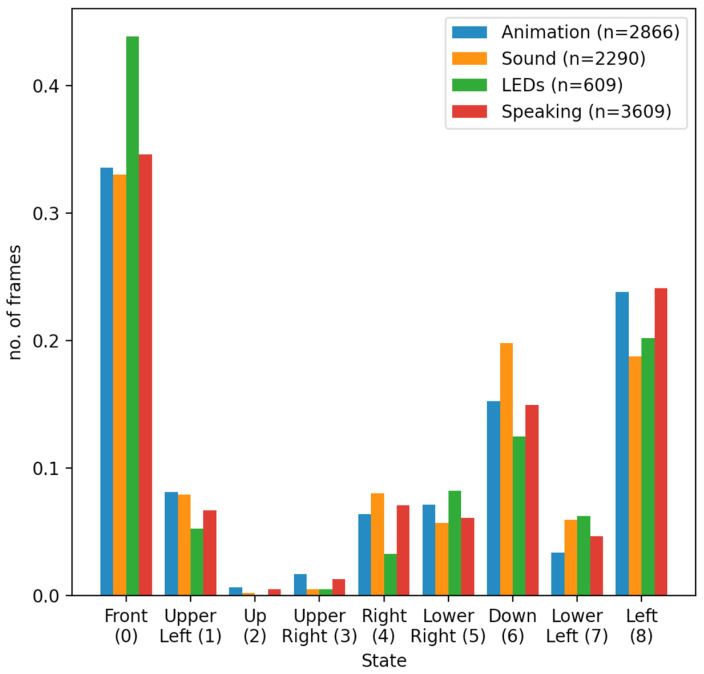
Four normalized distributions of the set of complete frames over the set of nine child states for four different robot modalities, namely Animation, Sound, LEDS and Speaking.

Figure 8 displays the four different normalized histograms of complete frames over the nine child states. Each histogram can be interpreted as a probability mass function induced from experimental data. Table 3 displays the corresponding probability mass function values.

It is clear from Figure 8 that the majority of frames belong in the Front state, regardless of the robot modality. The latter is a confirmation of the fact that the robot attracted the child’s attention when it was producing some audio or visual stimulus. In particular, the aforementioned effect appears to be more pronounced when the robot was performing an animation or speaking. It must be noted that robot speech was configured to also be accompanied by random hand gestures, and therefore, for this robot modality, both audio and visual stimuli were present.

The comparison between different robot modalities, for the specific 7 children that participated in the study, reveals that the LEDs tend to result in the child facing the robot more than any other robot stimuli and especially other robot-generated visual stimuli such as animations. This can be explained by two facts: (1) The LEDs are located in the eyes of the robots; therefore, this particular stimulus requires more direct eye contact, as opposed to alternative robot movements, which can be perceived even if the head is turned, and (2) LED stimuli occurred rather rarely during the sessions; therefore, they are more interesting when they happen. Note that the total number of frames in which active LEDs are involved is small (*n* = 609) compared to frames regarding Animation (*n* = 2866), Sound (*n* = 2290) or Speaking (*n* = 3609). For Sound and Speaking robot modalities head orientations other than Front seemed to be prevalent. This can be explained by considering that these two categories are based on audio stimuli, and so they do not require direct eye contact. Similar analyses can be conducted to study the effect of alternative robot modalities such as specific animations or sounds or the recitation of specific stories or instructions.

In the context of this work, LC emerges naturally in two different manners. First, a probability space constitutes a mathematical lattice equipped with a positive valuation function; the latter is the corresponding probability measure. Second, in the lattice of interval trees, a positive valuation function is also available. Recall that LC computes with semantics represented by the partial order relation.

Robot actions may be interpreted as “causes”, whereas behavioral data may be interpreted as the corresponding “effects” to the causes. By identifying, conditionally, a reliable functional relation from robot actions to human behaviors, actions of a social robot can be designed toward assisting a human expert in ASD interventions. Nevertheless, due to nature of human–robot interaction, the quest for a deterministic functional mapping from robot actions child states is not expected to be fruitful. Hence, a different functional mapping is sought as discussed below.

### 4.2. Events and Child’s State Transitions

Additional data of interest to clinical psychologists regard child response to an *event*; recall that the latter has been defined above as “the outset of a robot action”. More specifically, observation over multiple sessions may identify factors influencing endogenous attention versus exogenous attention.

The recorded time series of complete frames in all sessions were scanned in order to identify every instance where an event had occurred. A *state transition*, by definition, occurs when the child state previous to an event state is different than the one following the event. Symbolically, let *s_i_*, *i* ∈ {1, …, N} be a time series of states in a session; furthermore, let an event occur at time *k* ∈ {1, …, N}. Then, a state transition, by definition, occurs at time *k* if and only if *s_k_*_−1_ ≠ *s_k+_*_1_.

**Figure 9:**
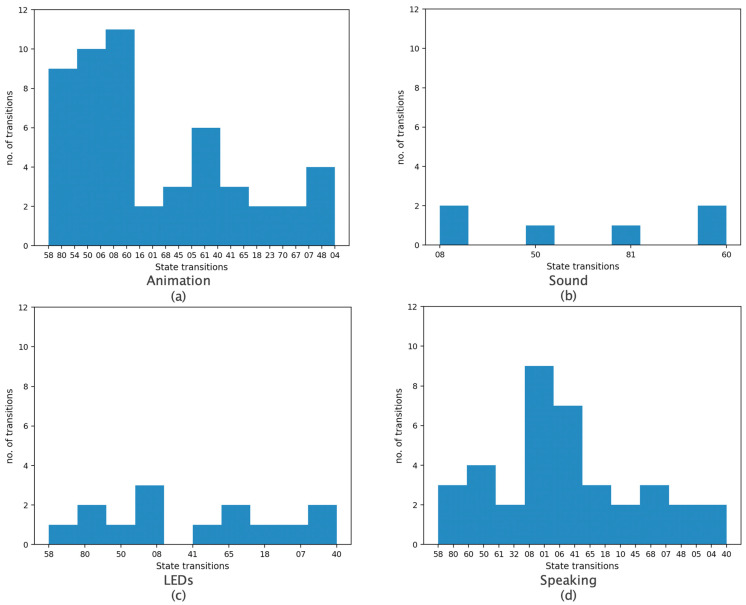
Histograms of all children’s state transitions for four robot modalities, namely (**a**) Animation, (**b**) Sound (**c**) LEDs and (**d**) Speaking by the robot.

Figure 9 displays all the recorded transitions that correspond to all four different robot modalities: Animation, Sound, LEDs and Speaking. More specifically, a two-digit number “*ab*”, where *a*,*b* ∈ {0, …, 8}, represents a transition from state “*a*” to state “*b*”. Each subplot therefore shows which specific state changes have occurred when some robot action was initiated, as well as the number of state change occurrences. For example, in Figure 9a, the change from state “6” to state “0” occurred 11 times when animation had been initiated. Note that only frames where there was a transition from one state to another were plotted, whereas frames where there was no state change were ignored. To account for a possible “time lag”, alternative definitions for a state transition were considered, in particular *s_k_*_−1_ ≠ *s_k+_*_2_ and *s_k_*_−1_ ≠ *s_k+_*_3_.

Table 4 displays the results in detail. It can be observed that cumulatively, for all four modalities, the majority of transitions involved state “0” (Front). This is a further indication that robot actions attract the child’s attention and are more likely to motivate the child to establish visual contact.

### 4.3. Visualization of States

**Figure 10:**
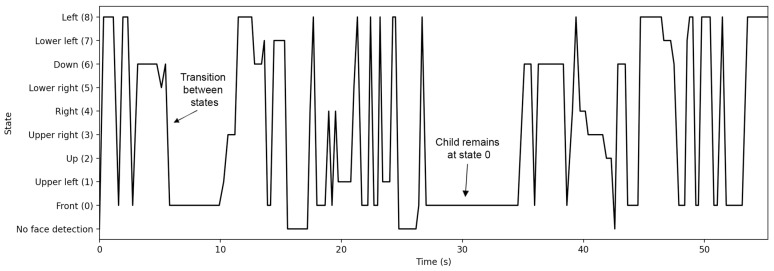
A child’s states during an eligible attempt that includes complete frames.

Figure 10 displays a time series of a child’s states during a session.

These were states that were detected during this sample session and were arranged in the order they appear, so that the progression of, and transitions between states during the scenario can be seen. Frames with no face detection, i.e., non-complete frames, are also shown. The visualization of the sequence of states during the execution of an activity can provide the researcher with clues regarding the predominant trends in child behaviors. For instance, the graph in Figure 10 may suggest the visual focus of attention and draw conclusions on sustained attention and the engagement level of the child.

### 4.4. Patterns of States

A child’s behavior can be considered to be a sequence of states. Frequent state sequences can reveal repeating patterns that, if they are observed to appear consistently after specific stimuli, can provide valuable clues to a clinical psychologist regarding the process. For example, does a child’s attention shift to the robot cue immediately or is the clinical psychologist’s involvement or aid needed? In addition, it can be studied whether similar robot actions (causes) trigger similar behaviors (effects).

To perform this type of analysis, the 90 attempts have been treated as 90 strings, each string containing of the successive states expressed as characters 0–8 as described above. With this notation, a pattern-matching algorithm was applied in order to identify any repeating patterns by assembling all the substrings contained at least 3 different states (which means that the substrings were at least of 3 characters in length). In addition, due to the sampling rate of 3 Hz, it was decided empirically that strings with up to 3 successive state repetitions are equivalent, and are therefore the same behavior executed at different speeds. For instance, the strings “806”, “8066”, “8806” and “80006” are equivalent. A set of equivalent patterns here is called *equivalence class*.

**Figure 11:**
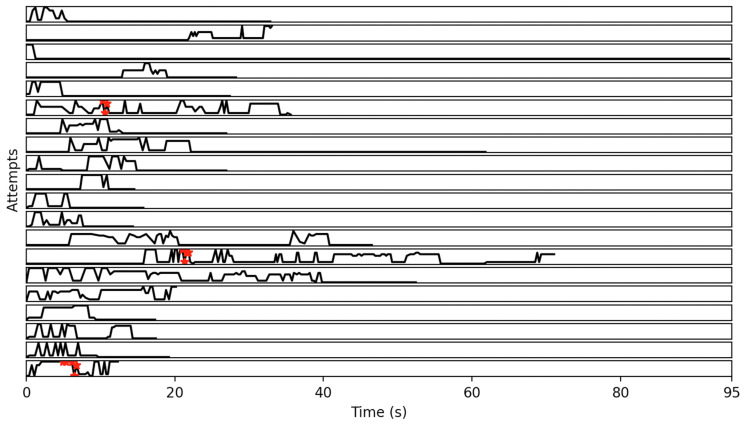
The occurrences of string “806”, as well as its equivalent strings, are indicated in red color in 20 eligible attempts, randomly selected.

The set of all strings with the aforementioned criteria were computed in all the 90 eligible attempts, and it was found that state sequence (string) “806” and its equivalent patterns occurred most frequently. More specifically, the aforementioned strings (and their frequencies within parentheses) include “806” (16), “8066” (11), “8806” (11), “8006” (11), “88806” (7), “80666” (7), “88066” (7), “80066” (7), “88006” (7), “888006” (6), “800666” (6), “880666” (5), “888066” (4), “880066” (3), “80006” (3), “8880066” (2), “880006” (2), “8800666” (2), “8880666” (2), “88800666” (1), “800066” (1), “8800066” (1), “8880006” (1), “88800066” (1). Figure 11 displays 20 attempts, randomly selected out of the 90 eligible attempts, that include complete frames. It also indicates the occurrences of equivalent to “806” strings in red color.

**Figure 12:**
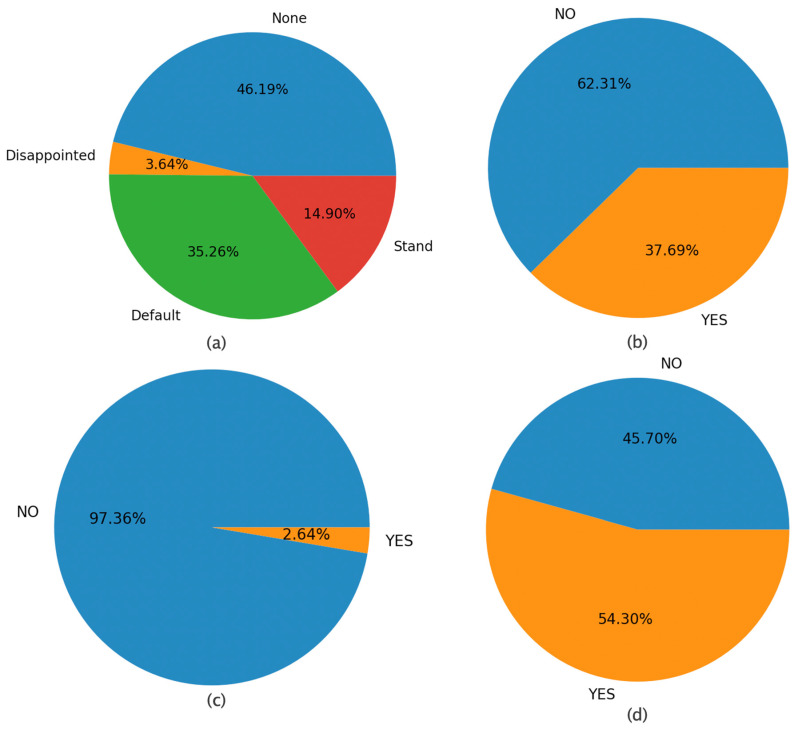
Distribution of the 604 complete frames in the equivalence class of the string “806” in all the 90 eligible attempts over each set of robot actions regarding four robot modalities, namely (**a**) Animation, (**b**) Sound, (**c**) LEDs and (**d**) Speaking.

In all, 124 occurrences of string “806”, as well as its equivalent strings, appear, including a total number of 604 complete frames distributed per robot modality values as follows: (a) Animation (None (279), Disappointed (22), Default (213), Stand (90)), (b) Sound (NO (377), YES (227)), (c) LEDs (NO (589), YES (15)), and (d) Speaking (NO (276), YES (328)) as shown in Figure 12, which displays the results in all 90 attempts. In particular, Figure 12 displays how the possible values of each modality are distributed, for the frames where the pattern “806” and equivalent patterns occur (i.e., for the particular equivalence class). It can be noted that the most frequent pattern “806” rarely appears with LEDs robot modality. The latter is explained by the fact that LEDs fully absorb a child’s attention. Furthermore, Figure 12 shows that the pattern “806” rather appears with either Speaking or Sound. The latter can suggest a pattern of interest to clinical psychologists for further study.

It has been observed that the most frequent equivalent patterns are similar, except for two major differences: first, the order of appearance of the states, and second, the number of repetitions of a state before the transition to a new state. The first difference indicates that certain state transitions are more common than others, regardless of the order of their appearance. By knowing what the states represent—in this case a head pose—the expert can identify the meaning of repeated state changes in behavioral terms. The second difference indicates that a pattern of states can have a variable duration because a child may remain in a particular state for some time as was explained above.

The developed software allows for the correlation of a particular pattern of states with the robot actions, i.e., to identify which robot actions were active for the duration of the state sequence in question. In this way, the expert can make assumptions on whether specific robot actions cause certain repeated behaviors. For example, the histograms of Figure 12 reveal the active robot actions when pattern ‘806′ occurred. In particular, it can be seen that the particular pattern of states occurred more frequently when the robot was speaking and the animation was set to the default mode, i.e., when there were random hand gestures while the robot was speaking.

Taken together, the above four tools are valuable to clinicians because they can provide information on the different visual attentional processes and the sub-components of attention: alerting, orienting and inhibition, making the design of individualized robot-assisted interventions easier and more productive. They could also facilitate the comparison of ASD children with typically developed children against various tasks of joint attention. In addition, cluster analysis could be valuable as an objective marker of the intervention progress. This preliminary work has exclusively considered clusters defined in the space of tree data structures representing facial points. However, due to the LC applicability, additional data types can be accommodated modularly in order to induce enhanced clusters for sophisticated decision making as discussed in the following section.

## 5. Discussion and Conclusions

This work has reported on an ongoing project, which studies the effectiveness of social robots as tools in non-invasive psycho-educational interventions including Autism Spectrum Disorder (ASD). The shorter-term objective is to develop missing tools for clinical psychologists to carry out behavioral data analysis. The longer-term objective is to develop a dependable robot assistant to clinical psychologists. In the aforementioned context, an analysis of human behavioral data is necessary toward enhancing, first, the understanding of human responses to robot actions and, second, both social skills and emotional regulation by improving the robot-assisted intervention. Data were acquired during real-world ASD interventions at Papageorgiou General Hospital (PGH) in Thessaloniki, Greece where a social robot was interacting with children and, at the same time, recording both the children’s state and its own. It must be noted that additional data have been recorded manually by clinical psychologists for future statistical hypothesis testing regarding the effectiveness of social robots as assistants. However, this work has used exclusively data acquired by sensors mounted on the robot. In particular, 6846 complete frames were recorded out of a total number of 41,911 recorded frames.

Human behavior was represented structurally as a time series of states, where a state was a cluster induced from all available data regarding seven children. By employing novel data representation methods based on the lattice computing paradigm, the collected data were transformed into a form suitable for further processing in order to establish a basic behavioral model. At this stage, the model considers only the child’s head pose as an indication of the child’s attention but can be extended to include other cues. Data representation and modeling allowed a series of analyses and visualizations that can provide useful insights on the relationship between robot actions and child behavior, with the aim of improving the therapeutic protocols by incorporating the social robot in the most beneficial manner. More specifically, four computational tools were developed for behavioral data analysis including (1) histograms of states per robot modality, (2) histograms of state transitions per robot modality, (3) a visualization of time series of states and (4) histograms of robot actions per robot modality. The proposed four computational tools provide clinical psychologists with useful cues as explained per case in Section 4.

In this context, robot actions may be interpreted as “causes”, whereas behavioral data may be interpreted as the ensuing “effects”. Therefore, the proposed behavioral analysis can allow psychologists to design robot-assisted interventions to induce the intended human behavior, with a maximal likelihood of occurrence, always with respect to the established ethics; for instance, a human behavior to be pursued might be characterized by an increased attention. Consequently, an interaction between a human and a robot can be pursued. Another contribution of the present work is the modeling of behavior which, although only facial data were considered in this work, has the potential of including more behavioral cues such as body pose, voice volume, contextual speech, etc. Integration of these other cues can be achieved uniformly using the existing LC-based mathematical instruments and still be used in the same behavioral analysis tools described in this paper. The analysis methodology followed is generic and not designed to apply exclusively to the specific activities examined in this paper and does not require human behavior annotation as is common in other works. The fact that the behavioral states are induced from the data and not directly measured or observed is the main reason why a direct numerical comparison with other engagement measurement methods (gaze tracking, facial expression recognition, etc.) is difficult, and only a qualitative comparison was made in the paper. Behavioral states’ computation is data-driven, and behavioral patterns emerge from what the robot perceives. This means that the analytical tools presented in this paper can give psychologists studying the data the opportunity to make observations regarding behaviors either at the individual or the scenario level.

In practice, the results presented in the current paper give clues as to the robot stimuli that attract the child’s attention most. From the analysis presented here, which involves all cumulatively gathered data for all children across all activities, animations and the use of LEDs appear to prompt children to turn their heads toward the robot more frequently than other modalities, which suggests an increase in engagement. This finding can lead to the modification of activities in subsequent sessions to include more animations and use of LEDs. Furthermore, the expert is able to isolate the data collected from specific children to perform similar analyses and determine the stimuli to which particular children are most responsive to, and therefore provide the capability of designing individualized interventions, focused on the needs of specific individuals, thereby improving the therapeutic sessions over time. Similarly, data from specific therapeutic activities can reveal whether the effectiveness of stimuli vary depending on the context of each activity, based on the analysis of behaviors that manifest during the selected activity.

Potential future work could pursue the following objectives: (1) “On-robot” data processing toward real-time modification of robot actions toward improving interaction with a human over time; (2) computation of clusters considering not only the number of clusters but also the cluster *size* [44]; (3) consideration of more data from more children and therapeutic sessions in order to increase confidence in the results. Moreover, it is the authors’ intention to increase the number of variables that define a child state since, apart from the recorded 68 facial landmarks points, additional human behavioral data have been recorded including eye contact, gaze, body pose, hand gestures, speech, voice volume and others. The inclusion of these other variables will result in a more complete and accurate description of the child’s state, and consequently will produce more meaningful emergent behaviors. Similarly, the robot actions can also be examined in a more analytical fashion; for example, the effects of robot speech with specific content or the differences between individual types of animation can be studied. Finally, because of the non-specificity of the approach, the behavioral analysis can be applied in other areas of special education, such as learning difficulties.

## Figures and Tables

**Table 1:** A frame with values recorded by NAO robot during a session.

Robot Actions per Modality	Child State
(a) Animation modality actions: None, Disappointed, Default (movements while speaking), Stand.	Points in child’s face (68 facial landmarks)
(b) Sound modality actions: no/yes	
(c) LEDS modality actions: no/yes	
(d) Speaking modality actions: no/yes	
Frame sampling time: Date and time	

**Table 2:** Classification results for four environmental conditions.

Distance/Lighting Condition	Classification Results (%)	
	Tree	Vector
40 cm/Normal	97.8	86.8
40 cm/Dim	78.4	66.7
1 m/Normal	97.0	84.2
1 m/Dim	74.6	46.7

**Table 3:** Probability masses over the states for four different robot modalities.

Robot Modalities	Front (0)	Upper Left (1)	Up (2)	Upper Right (3)	Right (4)	Lower Right (5)	Down (6)	Lower Left (7)	Left (8)
Animation	0.335	0.081	0.007	0.017	0.064	0.071	0.152	0.034	0.239
Sound	0.330	0.079	0.003	0.005	0.080	0.057	0.198	0.059	0.189
LEDs	0.438	0.052	0	0.005	0.032	0.082	0.125	0.062	0.204
Speaking	0.346	0.067	0.005	0.013	0.071	0.061	0.149	0.046	0.242

**Table 4:** Number of “state transitions” for three different definitions of a “state transition”.

	No. of State Transitions at					
Robot	*s_k+_*_1_		*s_k+_*_2_		*s_k+_*_3_	
Actions	Yes	No	Yes	No	Yes	No
Animation	52	109	64	93	73	83
Sound	6	5	3	7	4	5
LEDs	14	19	10	22	12	21
Speaking	37	87	44	78	51	71
TOTAL	109	220	121	200	140	180

## Data Availability

Not applicable.

## Appendix A

In this section, the activities considered for behavioral analysis are described. The first of the relaxation activities in Table A1 is described in more detail in order to give a better understanding of a scenario flow and interaction style.

**Table A1:** Activities considered in the behavioral analysis.

**Free Play**	The robot welcomes the child and invites him/her to start playing in the provided area. During the play the robot motivates the child.
**Role Playing—Symbolic Games**	The robot asks the child a series of questions, involving storytelling, role playing and creation of stories The robot describes and demonstrates using gestures appropriate behaviors in various everyday scenarios such as how to approach friends, in a birthday party or a restaurant
**Theory of Mind**	Mimics: the children perform gross motor imitation tasks managed by the robot exclusively, with different levels of difficulty
**Targeted Behavior or Emotion**	(a) Shared attention scenario: The robot asks the child to identify objects in the room (b) Memory game with cards. The robot gives instructions of how the game is played (c) “I spy” game, guided by the robot
**Relaxation**	1. Robot: “Now I want to show you the relaxation corner; there, we will listen to music and learn various tricks you can do when you are not feeling well. Help me find the relaxation corner. Find the green circle on the wall. When you find it, tell me: green circle”. 2. Expected child response: “green circle or circle”. 2a. If yes, then the robot says: “Very nice. Let’s go to the relaxation corner now.” (Accompanied by the therapist and continues the robot to 3). 2b. If not: After 1st failure of child to answer, the robot says: “I’m not sure I understood well. Find the green circle and tell me a green circle”. After 2nd failure of child to answer, the robot says “We have not yet found the relaxation corner. Let’s try again. Look for the green circle and say “green circle”. If there is success, then go to 2a. After 3rd child failure to answer, the robot says “It is not easy to find the relaxation corner. Let’s try next time.” (therapist intervenes) 3. Robot: “Slow breaths help us to be calm. Listen to the sound we make when we take breaths, something like this:” (robot plays back a normal breathing sound). “We can take quick breaths like this:” (robot plays back a fast-breathing sound) “and very slow breaths like this:” (robot plays back a slow-breathing sound). “But only slow breaths help us to calm down. To start the game with the breaths, say: “now”. 4. Expected child response: “now”. 4a. If yes, then the robot says: “Very nice. Let’s start the game with the breaths.” 4b. If not: - After 1st child failure to answer, the robot says: “I’m not sure I understood well. Say the word “now” to start the game with the breaths”. If there is success then 4a. - After 2nd child failure to answer, the robot says: “It seems that you are not sure that you want us to play the game with the breaths. We can try next time” (therapist intervention).
	The therapist gives the child a pinwheel, and the robot explains the exercise, which is that, at the instruction of the robot, the child must blow on the pinwheel in order to make it spin.
	Muscle relaxation scenario where the robot instructs the child in various exercises involving breathing and muscle relaxation.
